# [Retracted] Combined effects of Lenvatinib and iodine-131 on cell apoptosis in nasopharyngeal carcinoma through inducing endoplasmic reticulum stress

**DOI:** 10.3892/etm.2026.13274

**Published:** 2026-08-17

**Authors:** Guoyu Wang, Juhua Zhuang, Jing Ni, Ying Ye, Saifei He, Wei Xiai

Exp Ther Med 16:3325–3332, 2018; DOI: 10.3892/etm.2018.6652

Following the publication of the above paper, it was drawn to the Editor’s attention by a concerned reader that certain of the western blot data featured in Fig. 3A on p. 3330 had already been published in an article in the journal *International Journal of Molecular Sciences* that was written by different authors at different research institutes. A subsequent investigation of this paper undertaken by the Editorial Office also revealed that western blot data in Fig. 2D and immunohistochemical data in Fig. 3D had also been featured in other previously published articles that were written by different authors at different research institutes.

Owing to the fact that the contentious data in the above article had already been published prior to its submission to *Experimental* and *Therapeutic Medicine,* the Editor has decided that this paper should be retracted from the Journal. The authors were asked for an explanation to account for these concerns, but the Editorial Office did not receive a reply. The Editor apologizes to the readership for any inconvenience caused.

